# TGFß1 Stimulates Lymphatic Endothelial Cells to Produce IL7 and IL15, Which Act as Chemotactic Factors for Breast Cancer Cells with Mesenchymal Properties

**DOI:** 10.1007/s10911-023-09552-y

**Published:** 2023-12-06

**Authors:** Nikolina Giotopoulou, Wenyang Shi, Malgorzata Maria Parniewska, Wenwen Sun, Jonas Fuxe

**Affiliations:** 1 Department of Laboratory Medicine, Division of Pathology, Karolinska Institutet, Stockholm, SE-14152 Sweden; 2 Department of Oncology-Pathology, Karolinska Institutet, Stockholm, SE-17164 Sweden; 3 Division of Clinical Pathology and Cancer Diagnostics, Karolinska University Laboratory, Karolinska University Hospital, Stockholm, SE-14186 Sweden

**Keywords:** Lymph metastasis, Chemotactic migration, EMT, TGFβ1, IL7, IL7R, IL15, IL15RA

## Abstract

The lymphatic system is a major gateway for tumor cell dissemination but the mechanisms of how tumor cells gain access to lymphatic vessels are not completely understood. Breast cancer cells undergoing epithelial-mesenchymal transition (EMT) gain invasive and migratory properties. Overexpression of the cytokine transforming growth factor β1 (TGFβ1), a potent inducer of EMT, is frequently detected in the tumor microenvironment and correlates with invasion and lymph metastasis. Recently, we reported that TGFβ1 stimulated breast cancer cells with mesenchymal properties to migrate in a targeted fashion towards the lymphatic system via CCR7/CCL21-mediated chemotaxis, similar to dendritic cells during inflammation. Here, we aimed to identify additional chemotactic factors and corresponding receptors that could be involved in guiding breast cancer cells through the lymphatic system. Through a combination of RNA sequencing analysis, database screening and invasion assays we identified IL7/IL7R and IL15/IL15R as pairs of chemokines and receptors with potential roles in promoting chemotactic migration of breast cancer cells with mesenchymal properties towards the lymphatics. The results demonstrate the capacity of TGFβ1 to orchestrate crosstalk between tumor cells and lymphatic endothelial cells and warrant further studies to explore the roles of IL7 and IL15 in promoting lymph metastasis of breast cancer.

**Supplementary Information:**

The online version contains supplementary material available at 10.1007/s10911-023-09552-y.

## Introduction

During inflammation, dendritic cells are activated and migrate to lymph nodes, where they perform antigen presentation to T cells. Migration of dendritic cells through the lymphatic system occurs in a targeted fashion and is driven by chemotaxis. The mechanism involves upregulation of chemokine receptors like CCR7 in activated dendritic cells allowing them to sense and migrate towards lymphatic endothelial cells expressing CCL21. Endothelial cells of lymphatic capillaries are equipped with semi-open, button-like junctions, that support uptake of cells and fluid into the lymphatic system [1]. Thus, the step of intravasation of cells into the lymphatic system is supported by structural components in lymphatic capillaries.

Lymph metastasis is an important prognostic factor and usually the first sign of metastatic spread in breast cancer and other types of cancer [2]. Breast cancer patients with lymph node metastasis have a 40% lower five-year survival rate compared to those without [3]. Despite its clinical relevance, the underlying mechanisms driving tumor cell dissemination through the lymphatic system remain incompletely understood. Breast cancer cells acquire invasive and migratory properties by undergoing epithelial-mesenchymal transition (EMT) [4, 5]. EMT is a latent developmental process, which can be reactivated in cancer tissues where cytokines with EMT-inducing properties are frequently overexpressed. Thus, EMT is associated with and to a certain degree, driven by tumor-related inflammation [6]. Overexpression of the cytokine TGFβ1, a potent inducer of EMT, in breast cancer tissues and plasma samples from breast cancer patients is associated with lymph node metastasis [7, 8]. TGFβ1-induced EMT is driven by transcriptional reprogramming and involves cooperative action of Smad transcription factors and core EMT-TFs including members of the SNAIL, ZEB and TWIST families [9, 10].

Tumor cells undergoing EMT lose epithelial characteristics including apical-basal polarity and cell-cell junctions. In parallel, they gain mesenchymal features including an elongated, fibroblast-like morphology, a reorganized cytoskeleton, and invasive and migratory capabilities. EMT is a plastic process and cells may shift between different epithelial/mesenchymal states during the metastatic process. The plasticity of EMT is underscored by studies showing that breast cancer cells undergoing EMT adopt properties of mesenchymal stem cells and have the capacity to transdifferentiate into adipocytes [11, 12].

Previously, we reported that TGFβ1-induced EMT promotes lymphatic dissemination of breast cancer cells by activating CCL21/CCR7-mediated chemotaxis [13, 14]. This is in line with studies showing the involvement of CCR7 and CXCR4 in breast cancer metastasis [15, 16]. Yet, recent reports indicate that the role of CCR7 seems to vary between different subtypes of breast cancer [17], and that expression of CCR7 in breast cancer tissues is not predictive for lymph metastasis [18]. Based on this, we set out to explore possible additional chemotactic mechanisms linking TGFβ1 to EMT and lymphatic dissemination of breast cancer cells.

## Materials and Methods

### Cell Culture

Namru Murine Mammary gland (NMuMG) cells were from ATCC (American Type Culture Collection, VA) and cultured in DMEM (Thermo Fisher Scientific, Gothenburg, Sweden) containing high glucose (4500 g/ml) and supplemented with 10% Fetal Bovine Serum (FBS, Thermo Fisher Scientific) and 1% penicillin + streptomycin. EpXT cells were provided from the laboratory of by Hartmund Beug (Vienna Medical University), and were cultured in DMEM/F-12 medium (Thermo Fisher Scientific) supplemented with 10% FBS and 1% penicillin + streptomycin. MDA-MB-231 cells (ATCC) were cultured in DMEM (Thermo Fisher Scientific) supplemented with 10% FBS and 1% penicillin–streptomycin. Mouse lymphatic endothelial cells (SV-LEC) [19] (kindly provided by Dr Jonathan S. Alexander, Louisiana State University Health Sciences Center, Shreveport, Louisiana) were cultured in DMEM with high glucose supplemented with 10% FBS and 1% penicillin–streptomycin. Telomerase-immmortalized human dermal lymphatic endothelial cells (iLEC) [20] were kindly provided by Dr. Professor Lena Claesson Welsh at Uppsala University) and were cultured as previously described [14]. All cells were cultured in 37 °C and 5% humidified CO_2_ incubator.

For collection of conditioned medium from SV-LEC and iLEC cells, cells were seeded in 10 cm dishes (Thermo Fisher) and grown in medium containing low serum concentration (0.2%) and either supplemented or not with 10 ng/ml TGFβ1 (#P04202, R&D Systems, Abingdon, UK) and collected for 72 h. Control medium was generated in the same way using 10 cm dishes, in which no cells were seeded.

### Western Blot Analysis

Western blot was performed according to standard procedures, as described previously [21]. Primary antibodies used were: ZEB1 (AMAb90510, Atlas Antibodies, Lund, Sweden); N-cadherin (Ab98952, Abcam); IL7R (17626-1-AP-20, Proteintech); E-cadherin (3195 S, Cell Signaling); Vimentin (HPA001762, Atlas Antibodies); IL15RA (sc-374,023, Santa Cruz). Antibodies were selected for their documented or based on sequence similarity expected capacity to react with both mouse and human proteins.

### RNA Sequencing and Differential Expression Analysis

RNA sequencing analysis was performed at the core facility for Bioinformatics and Expression Analysis (BEA) at Karolinska Institutet. Total RNA was extracted from quadruplicate samples (technical replicates) of svLEC cells that were either left untreated or treated for 72 h with 10 ng/ml TGFβ1 using RNeasy Mini Kit (Qiagen, Valencia, CA) according to the manufacturer´s protocol. Libraries were prepared using TruSeq Stranded mRNA kit (Illumina) and sequenced on the Illumina Nextseq 550 platform, at single end mode and generating 75 bp reads that were mapped to the mouse reference genome (GRCm38/mm10) and Differential Expression Analysis was performed using EdgeR and the generalized linear model (GLM) likelihood ratio statistical test [22]. Threshold for significantly changing genes was set at FDR < 0.05. Gene Ontology Enrichment Analysis was performed in DAVID [23, 24] using significantly upregulated genes with a logFC > 1.5. The RNA sequence data have been submitted to Gene Expression Omnibus (GEO), accession number GSE241881.

### Quantitative Real-Time RT–PCR Analysis

The extraction of total RNA was done by using the RNeasy mini kit (Qiagen) according to the manufacturer’s instructions. cDNA synthesis was done by the use of QuantiTect Reverse Transcription kit (Qiagen) using an amount of 1 µg of total RNA. For qPCR (quantitative realtime PCR) analysis, cDNA mixture was used in an amount of 5 ng for PCR amplification by QuantiTect SYBR Green PCR Kit (Qiagen) with validated QuantiTect primers (Qiagen). The following genes were analyzed: Mouse *Il6*, *Il7*, *Il15*, *Cx3cl1*, *Serpine1*, *Cxcl16*, *Ccl17* and *l19;* Human *IL7* and *IL15*. The PCR was carried out as follows: 3 min at 95 °C followed by 35 cycles of 3 s at 95ºC, 20 s at 55 °C and 2 s extension step at 72 °C in Biorad PCR system. The sequence of used primers are listed in Table S1.

### Invasion Assays

Invasion assays were performed by using 8-µm pore cell culture inserts (Merck-Millipore, Solna, Sweden). Cloning cylinders (6 mm diameter, Thermo Fischer Scientific) were placed in the inserts and 200 µl of growth factor-reduced Matrigel (3 mg/ml, diluted 1:10 in DMEM medium, AH Diagnostics, Solna, Sweden) was added into the cell culture inserts and allowed to solidify for 1 h in a 37 °C incubator. 30,000 cells (in 50 µl DMEM containing no FBS) were seeded on top of Matrigel plugs in the culture inserts. NMuMG cells were pre-treated with 10ng/ml of TGFβ1 for 72 h to induce EMT, while EpXT cells and MDA-MB-231 cells were untreated. Inserts containing cells seeded on top of Matrigel plugs were subsequently placed in separate wells of a 24-well plate, in which 700 µl of control medium (DMEM without FBS), control medium supplemented with recombinant TNFa (10 ng/ml), IL7 (10ng/ml) or IL15 (10ng/ml) (#P01375; #P13232.1; #P40933.1; all from R&D Systems), or conditioned medium collected during 72 h from untreated or TGFβ1-treated (10 ng/ml) SV-LECs had been added. A neutralizing IL7 antibody (#PA5-46944, Thermo Fisher Scientific), or an isotype matched control antibody (#AB-108-C, Biotechne Ireland Limited, Dublin, Ireland), was added to the conditioned medium from TGFβ1-treated SV-LECs at a concentration of 80 ng/ml 30 min prior to the invasion assays. After 4 h, cells that had migrated to the lower chamber were counted using Alamar blue and Countess (Thermo Fisher Scientific) for three separate measurements in each of the triplicates of each sample.

### Cytokine Analysis

The cell culture medium of svLEC stimulated by the TGFβ1 (10 ng/ml) for 72 h was tested by enzyme-linked immunosorbent assay (ELISA) kit for detection of the concentration of mouse IL7 (Proteintech, KE10028) and IL5 (Proteintech, KE10060). The supernatants were centrifuged to remove cellular debris and concentrated by Amicon Ultra-2 Centrifugal Filter Unit (Merk). The experiment was performed in triplicate.

### Statistical Analysis

Statistical analysis for comaprisons between two groups were performed with unpaired T-test. For comparisonsn between more than two groups, ANOVA was used followed by Tukey’s multiple comparisons test using Prism 9. Data represent means ± s.e.m. with three independent experiments in triplicate. The following significance levels were used: *P < 0.05; **P < 0.01; ***P < 0.001; ****P < 0.0001.

## Results

### Breast Cancer Cells Undergoing TGFβ1-Induced EMT Acquire Chemotactic Properties

**Fig. 1:**
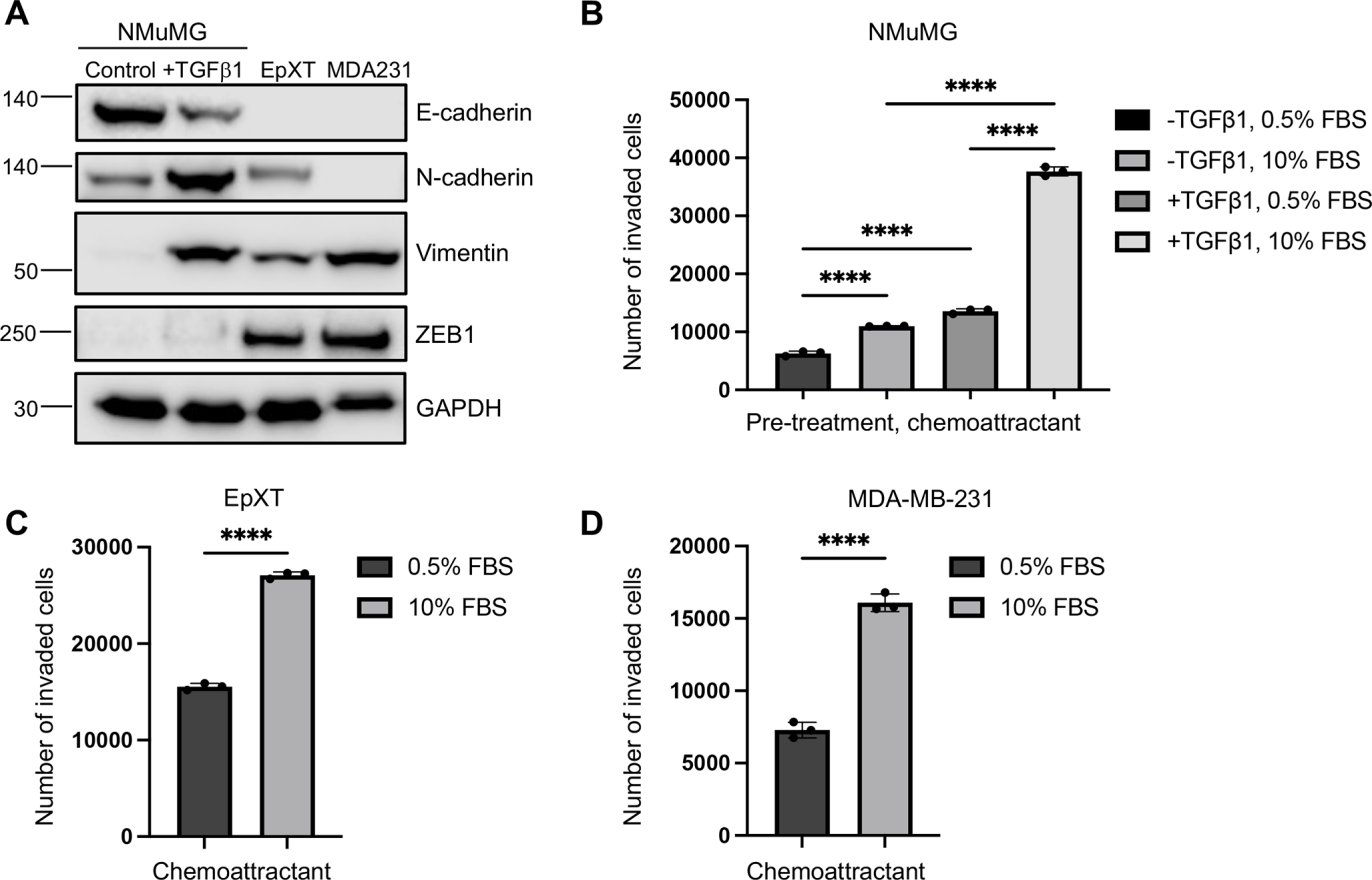
Chemotactic properties of breast cancer cells with mesenchymal characteristics. (**A**) Representative western blot of EMT markers in non-treated (control) and TGFβ1 treated (10 ng/ml, 72 h) NMuMG cells, EpXT cells and MDA-MB-231 (MDA231) cells. E-cadherin, N-cadherin, ZEB1 and vimentin were used as EMT markers. GAPDH was used as a loading control. (**B**) Bar graph showing results from invasion assays, in which the effects of pre-treatment with TGFβ1 (10 ng/ml, 72 h) and/or adding 10% FBS as a chemoattractant in the lower chamber on the invasive capacity of NMuMG cells was analyzed. (**C, D**) Bar graphs showing results from invasion assays analyzing the effect of adding 10% FBS as a chemoattractant in the lower chamber on the invasive capacity of EpXT cells (**C**) and MDA-MB-231 cells (**D**). Data represent mean results from three independent experiments with three technical replicates per condition and experiment. **** = P < 0.0001

A combination of cellular models was used to study chemotactic properties of breast cancer cells with mesenchymal characteristics. Namru mouse mammary gland (NMuMG) epithelial cells are frequently used as a model of transient, TGFβ1-induced EMT [10, 25]. Mouse mammary EpXT tumor cells, on the other hand, is continuously in EMT through cooperative effects of TGFβ1 and oncogenic Ras signaling [26]. Human MDA-MB-231 breast cancer cells are classified as basal-like breast cancer cells with mesenchymal properties [27]. Western blot analysis with the commonly used EMT markers E-cadherin, N-cadherin, vimentin and ZEB1 demonstrated that NMuMG cells exposed to TGFβ1 (10ng/ml, 72 h), acquired EMT characteristics and confirmed mesenchymal properties of EpXT cells and MDA-MB-231 cells (Fig. 1A and Supplementary Fig. S1A-D).

Invasion assays were used to study the impact of TGFβ1-induced EMT on the chemotactic properties of NMuMG cells. NMuMG cells that had been induced to undergo EMT by exposure to TGFβ1 invaded slightly more efficiently compared to non-treated cells when medium with 0.5% fetal bovine serum (FBS) was used both in the upper and lower chambers (Fig. 1B). However, when medium with high concentration of FBS (10%) was added to the lower chamber as a chemoattractant, TGFβ1-treated cells migrated significantly more efficiently compared to non-treated cells. The results indicated that NMuMG cells undergoing TGFβ1-induced EMT acquired enhanced chemotactic properties and the ability to sense and migrate towards factors present in FBS. Invasion assays with 10% FBS as a chemoattractant were also performed with EpXT and MDA-MB-231 cells and the results showed that these cells invaded more efficiently in the presence of 10% FBS relative to 0.5% FBS in the lower chamber (Fig. 1C, D).

### Lymphatic Endothelial Cells Exposed to TGFβ1 Secrete Factors that are Chemotactic for Breast Cancer Cells with Mesenchymal Properties

**Fig. 2:**
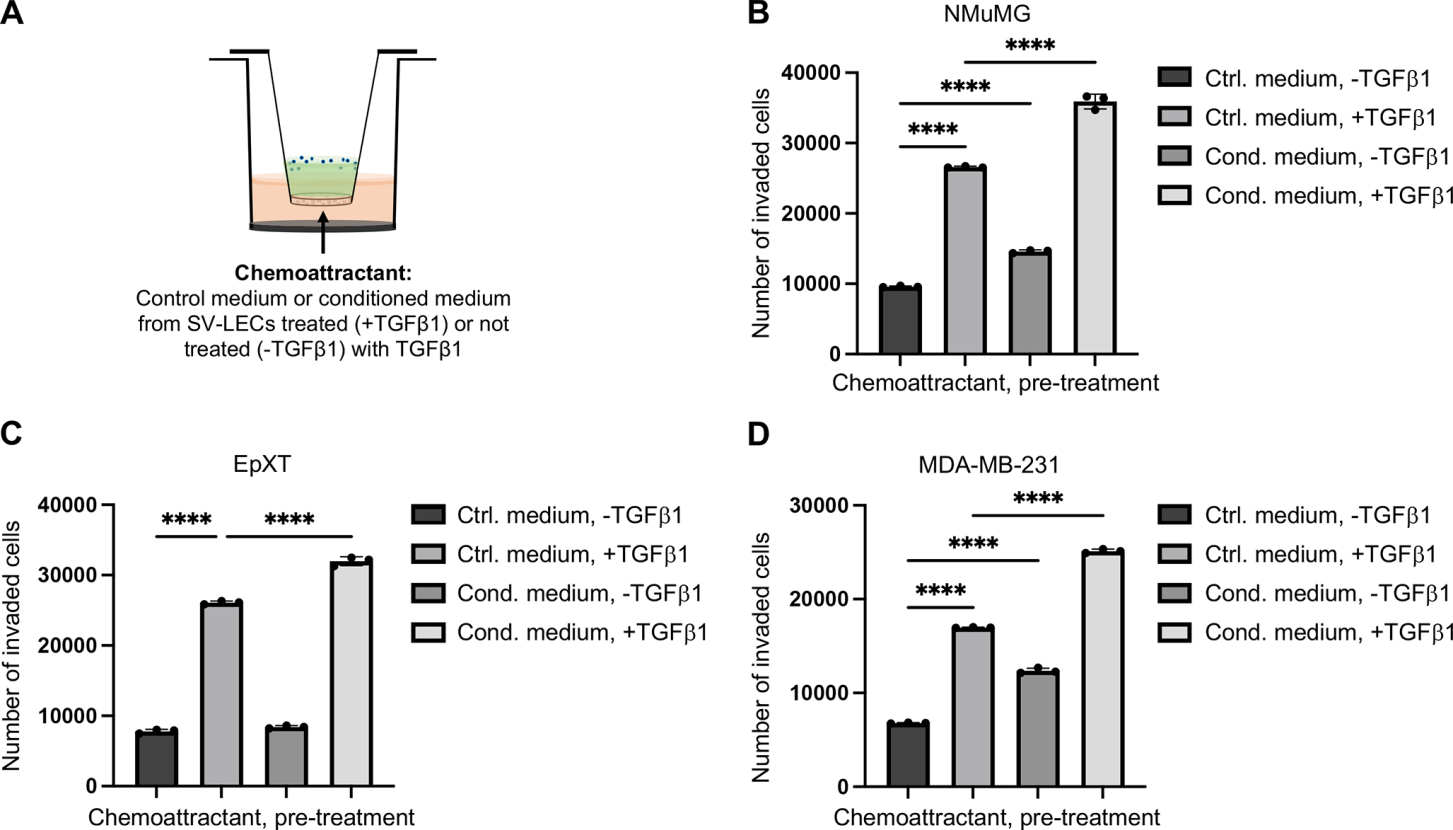
Lymphatic endothelial cells exposed to TGFβ1 secrete factors that promote invasion of breast cancer cells with mesenchymal properties. (**A**) Schematic drawing of the experimental setup where either control (Ctrl.) or conditioned (Cond.) medium from untreated (-TGFβ1) or TGFβ1-treated (+ TGFβ1) SV-LEC cells was used as a chemoattractant in invasion assays. (**B-D**) Bar graphs showing results from invasion assays, in which the effects of control medium or conditioned medium from untreated or pre-treated with TGFβ1 (10 ng/ml, 72 h) SV-LECs on the invasive capacity of NMuMG cells (**B**), EpXT cells (**C**) and MDA-MB-231 cells (**D**) were analyzed. Data represent mean results from three independent experiments with three technical replicates per condition and experiment. **** = P < 0.0001

As a next step, invasion assays were performed to study whether TGFβ1 could stimulate lymphatic endothelial cells (SV-LEC) to secrete chemoattractants for breast cancer cells with mesenchymal properties. Control medium or conditioned medium from SV-LEC lymphatic endothelial cells that had been left untreated or treated with TGFβ1 (10 ng/ml) for 72 h was collected and used in invasion assays (Fig. 2A). The results showed that conditioned medium from TGFβ1-treated SV-LECs increased the invasive capacity of NMuMG cells significantly more efficient than conditioned medium from untreated SV-LECs, or control medium (Fig. 2B). Similar results were found for EpXT cells and MDA-MB-231 cells, showing that these cells migrated more efficiently towards conditioned medium from TGFβ1-treated SV-LEC cells compared to conditioned medium from untreated SV-LECs, or control medium (Fig. 2C, D). The results indicated that TGFβ1 stimulates SV-LECs to produce chemotactic factors that promote invasion of breast cancer cells with mesenchymal properties.

### RNA Sequencing Identifies Chemotactic Factors Induced in Lymphatic Endothelial Cells Exposed to TGFβ1

**Fig. 3:**
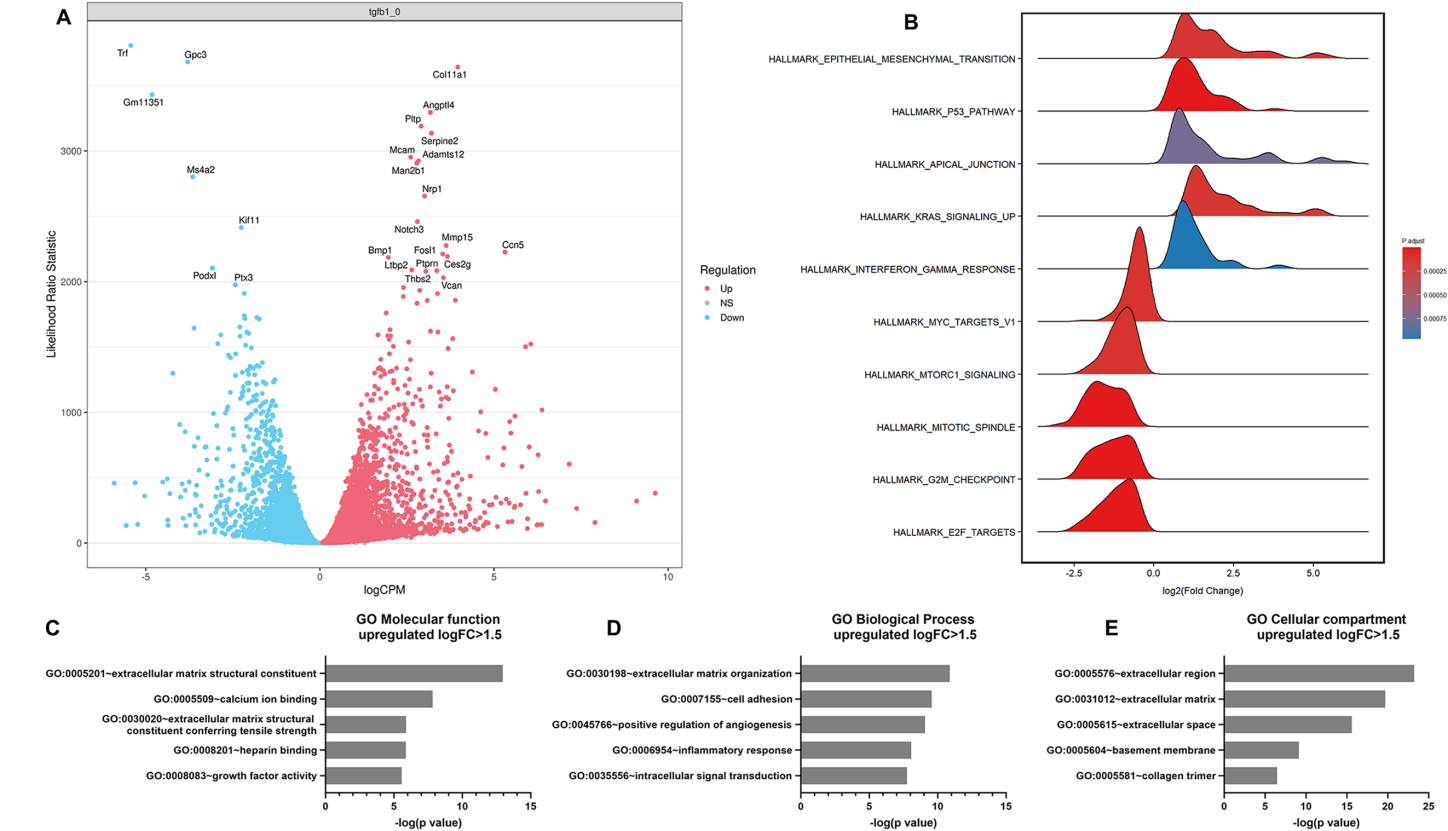
RNA seq analysis identifies TGFβ1-induced genes in SV-LECs. (**A**) Volcano plot showing differentially expressed genes in SV-LEC lymphatic endothelial cells treated with TGFβ1 versus non-treated cells. Red and blue dots indicate significantly 4971 upregulated and 4996 downregulated genes, respectively, among a toal of 12,869 mRNAs that were detected. (**B**) Gene set Enrichment analysis of molecular signature database Hallmark gene sets. (**C-E**) Analysis of top upregulated genes in SV-LEC cells after TGFβ1 treatment. Top 5 Gene Ontology terms enriched among top upregulated genes (logFC > 1.5) with respect to Molecular Function (**D**) Biological Process (**E**), and Cellular Compartment (**F**) are shown

To identify chemotactic factors induced in SV-LECs by TGFβ1 that could stimulate chemotaxis of breast cancer cells with mesenchymal properties we performed RNA sequencing and differential gene expression analysis of TGFβ1-treated versus non-treated SV-LECs. Global expression analysis showed that out of 12,869 mRNAs detected, 4971 were significantly upregulated while 4996 genes were significantly downregulated in SV-LECs after exposure to TGFβ1 (Fig. 3A). Hallmark enrichment analysis showed that EMT-related genes were at the top of the induced genes, while genes involved in cell cycle regulation (E2F_TARGETS; G2M_CHECKPOINT) were among the most downregulated ones (Fig. 3B). Pathway analysis using the Kyoto Ecoclopedia of Genes and Genomes (KEGG) database showed that induced genes were involved in focal adhesion, cytokine- and extracellular matrix-mediated receptor interactions and that repressed genes were involved in cell cycle regulation (Supplementary Fig. S2A).

To narrow down our search for chemotactic factors induced in SV-LECs by TGFβ1 we focused on 685 genes significantly upregulated by logFC > 1.5. Gene ontology analysis showed that these genes encoded proteins with various molecular functions and biological processes including extracellular matrix organization, calcium ion binding, heparin binding, growth factor activity, cell adhesion, angiogenesis and inflammation (Fig. 3C, D). Further analysis using the term “cellular compartment” identified a cluster of 120 induced genes encoding proteins secreted into the extracellular space (Fig. 3E, Supplementary Table S2).

**Fig. 4:**
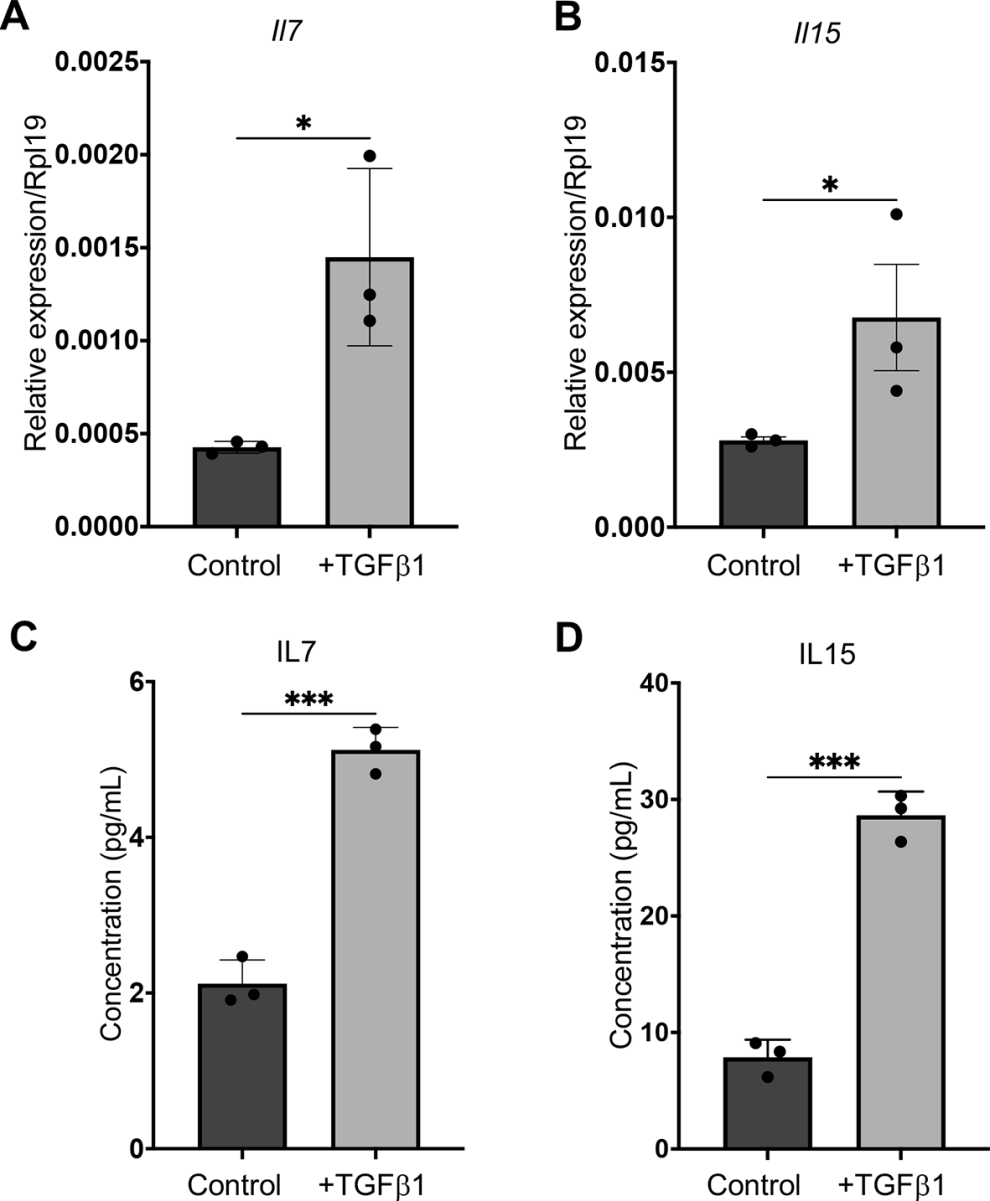
Induction of *IL7* and *IL15* in SV-LEC cells by TGFβ1. (**A, B**) QPCR results validating the RNA seq data showing induction of *Il7* and *Il15* in TGFβ1 treated compared to non-treated SV-LEC cells. (**C, D**) Results from ELISA assays showing increased IL7 and IL15 levels in conditioned medium from SV-LEC cells exposed to TGFβ1 compared to control. Data represent mean results from three independent experiments with three technical replicates per condition and experiment. *** = P < 0.001; * = P < 0.05

Within this cluster, we identified three chemokines: *Cx3cl1*, *Cxcl16* and *Ccl17*; and three members of the interleukin family: *Il7*, *Il15* and *Il6*, among the TGFβ1-induced genes encoding extracellular proteins in SV-LECs (Supplementary Fig. S2B). Of these, upregulation of *Il7* and *Il15* (Fig. 4A, B**)**, as well as *Ccl17* and *IL6* (Supplementary Fig. S3A-D) was confirmed by qPCR. To study if the induction of *Il7* and *Il15* in lymphatic endothelial cells was species-specific we exposed human immortalized lymphatic endothelial cells (iLEC) to TGFβ1 (10ng/ml, 72 h) and analyzed the expression of *IL7* and *IL15*. We found that *IL7* and *IL15* were upregulated also in iLEC cells by TGFβ1 (Supplementary Fig. S3E, F). Moreover, conditioned medium from TGFβ1-treated iLEC cells stimulated migration of MDA-MB-231 cells more efficiently compared to medium from non-treated iLECs (Supplementary Fig. S3G). The results suggested that IL7 and IL15 could function as chemotactic factors for breast cancer cells with mesenchymal properties. To explore this we studied whether the upregulation of *IL7* and *IL15* in SV-LEC by TGFβ1 resulted in increased secretion of these interleukins. We analyzed conditioned medium from TGFβ1-treated versus non-treated SV-LEC cells by ELISA and found that the concentration of both IL7 and IL15 was increased in the medium from TGFβ1-treated compared to non-treated SV-LEC cells (Fig. 4C, D).

**Fig. 5:**
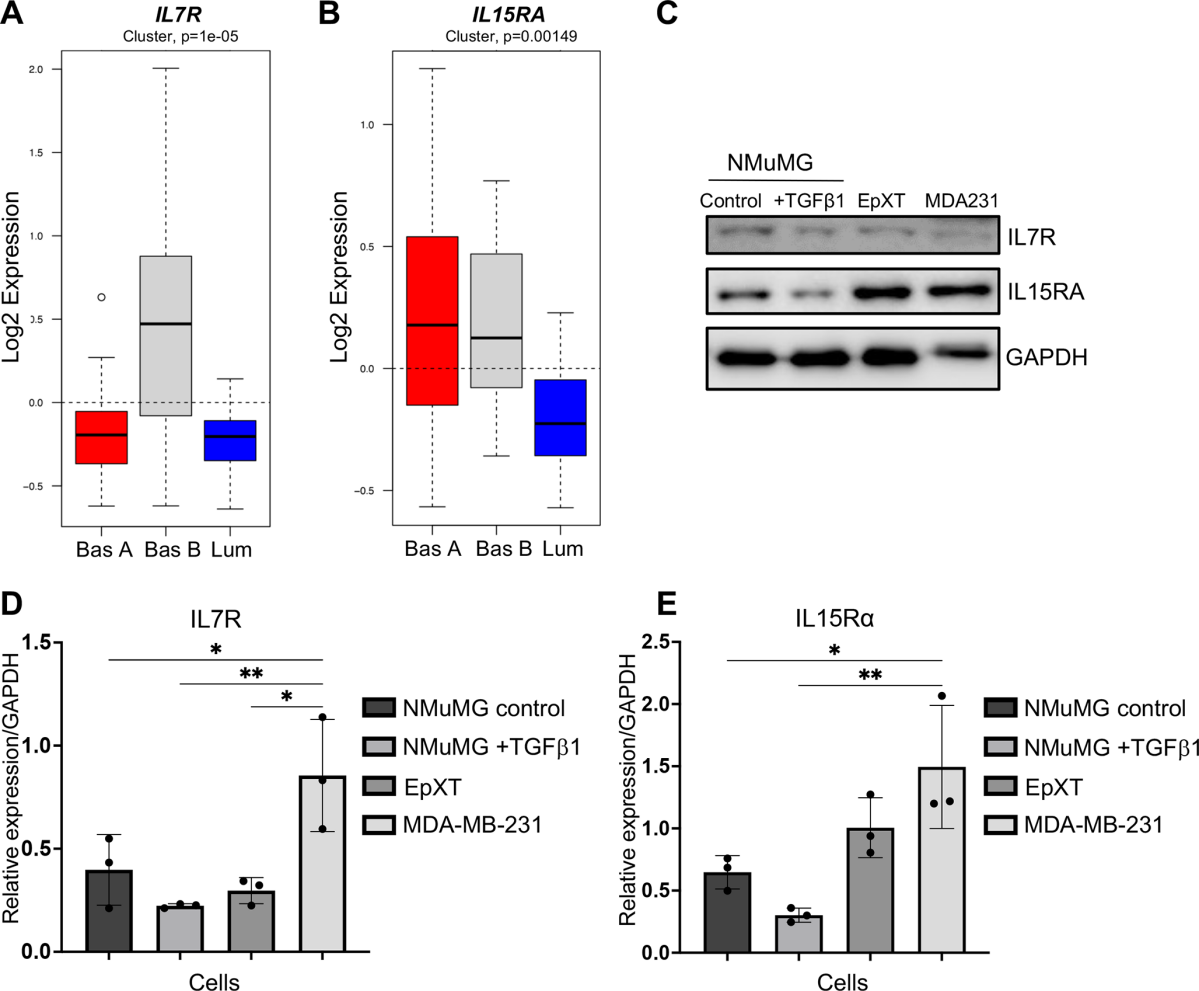
Expression of IL7R and IL15RA in subtypes of breast cancer cells. (**A, B**) Log2 expression data from the GOBO database showing differences in the expression of *IL7R* (**A**) and *IL15RA* (**B**) in human breast cancer cells classified as basal B (Bas B) cells, which are known to display mesenchymal properties, compared to luminal (Lum) cells. (**C**) Representative results from western blot analysis of IL7R and IL15RA protein expression in non-treated (control) and TGFβ1 treated NMuMG cells, EpXT cells and MDA-MB-231 (MDA231) cells. (**D, E**) Bar graphs showing quantification of IL7R (**D**) and IL15RA (**E**) protein levels from three independent Western blot experiments

This indicated that IL7 and IL15 could act as chemoattractants for breast cancer cells with mesenchymal properties. To evaluate this, we used the Gene expression-based Outcome for Breast cancer Online (GOBO) database to study whether the expression of their corresponding receptors was associated with mesenchymal properties in breast cells. The GOBO database contains gene profiling data of 51 human breast cancer cell lines that have been classified into luminal, basal A and basal B cells [28]. Among these, basal B cells are specifically known to display mesenchymal characteristics. The screening results showed that none of the receptors for Cx3cl1 (*CX3CR1*), Cxcl16 (*CXCR6*), Ccl17 (*CCR4*, *DARC*, *CCR10*) or IL6 (*IL6R*) was more expressed in basal B compared to basal A or luminal breast cancer cells (Supplementary Fig. S4A-F). In contrast, the receptors for IL7 (*IL7R*) and IL15 (*IL15RA*) were significantly overexpressed in basal B compared to luminal breast cancer cells (Fig. 5A, B and Supplementary Fig. S5A, B). The *IL7R* was specifically overexpressed in basal B cells while *IL15RA* was also overexpressed in basal A cells compared to luminal cells. In comparison, we found that the tumor necrosis factor receptor superfamily member 1 A (*TNFRSF1A*), a receptor for the cytokine TNFa, which is linked to EMT and metastasis in breast cancer [29, 30], was overexpressed in basal B cells (Supplementary Fig. S5C). Western blot analysis confirmed the expression of IL7R and IL15RA in MDA-MB-231 cells, and showed that these receptors were expressed also in NMuMG and EpXT cells (Fig. 5C-E), although the expression of both receptors appeared slightly lower in TGFβ1-treated NMuMG cells.

### IL7 and IL15 are Chemotactic Factors for Breast Cancer Cells with Mesenchymal Properties

**Fig. 6:**
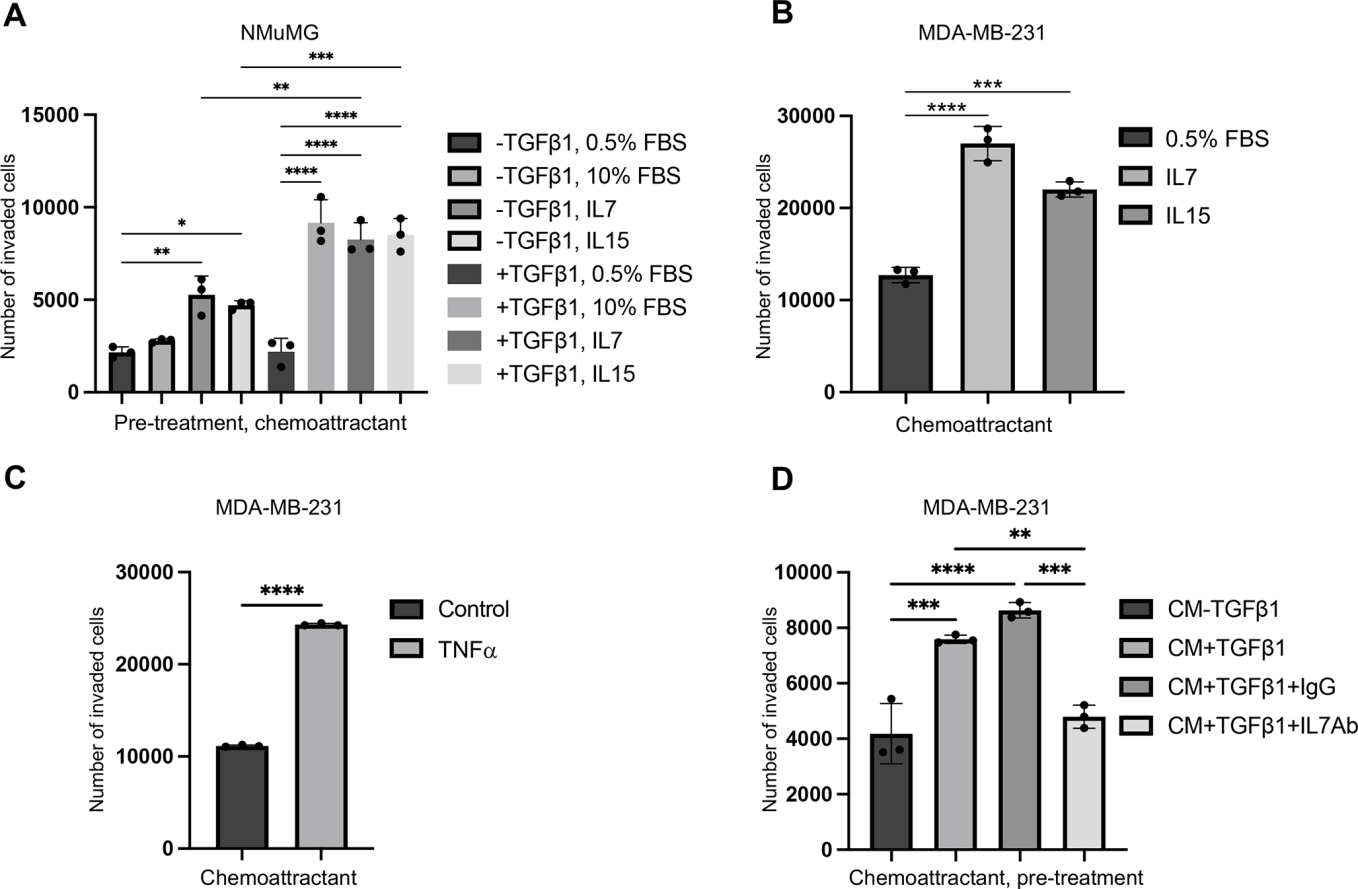
IL7 and IL15 act as chemoattractants for breast cancer cells with mesenchymal properties. (**A**) Results from invasion assays showing the capacity of IL7 (10 ng/ml) and IL15 (10 ng/ml) to promote migration of NMuMG cells pre-treated or not treated with TGFβ1. (**B, C**) Results from invasion assays showing that IL7 and IL15 (**B**) as well as TNFa (10 ng/ml) (**C**) stimulate migration of MDA-MB-231 cells more than control (0.5% FBS). (**D**) Results from invasion assays showing the effect of conditioned medium (CM) from untreated (-TGFβ1) or TGFβ1-treated (10 ng/ml, 72 h) SV-LECs on the invasion of MDA-MB-231 cells in the presence of a neutralizing antibody against IL7 (IL7Ab), or an isotype control IgG antibody (IgG). Data represent mean results from three independent experiments with three technical replicates per condition and experiment. **** = P < 0.0001; *** = P < 0.001; ** = P < 0.01; * = P < 0.05

Further studies using invasion assays were performed to evaluate whether adding either recombinant IL7 (10 ng/ml) or IL15 (10 ng/ml) as a single factor in the lower chamber could stimulate the invasive capacity of NMuMG and MDA-MB-231 cells. The results showed that each of IL7 and IL15 enhanced invasion of non-treated NMuMG cells compared to control (Fig. 6A). The stimulatory effect of IL7 and IL15 was more evident when mesenchymal NMuMG cells that had been pretreated with TGFβ1 were used in the assay and was similar to 10% FBS. IL7 and IL15 also stimulated the invasion of MDA-MB-231 cells significantly more efficient than 0.5% FBS (Fig. 6B). Similar to this, we found that also TNFα also stimulated invasion of MDA-MB-231 cells (Fig. 6C)

Together, the data suggested that IL7 produced and secreted by lymphatic endothelial cells exposed to TGFβ1 can act as a chemotactic factor for breast cancer cells with mesenchymal properties. To test this, we performed invasion assays using conditioned medium from SV-LEC cells exposed to TGFβ1 in the absence or presence of a neutralizing antibody to IL7. The results showed that adding a neutralizing antibody against IL7, but not a control IgG antibody, was sufficient to reduce the capacity of conditioned medium from TGFβ1-treated SV-LEC cells to promote invasion of MDA-MB-231 cells (Fig. 6D).

## Discussion

In this study, we combined cellular models of TGFβ1-induced EMT with RNA sequencing analysis, database screening and invasion assays to identify chemotactic mechanisms linking TGFβ1 to EMT and lymphatic dissemination of breast cancer cells. The results showed that cells undergoing TGFβ1-induced EMT acquire significantly enhanced chemotactic capabilities. This indicates that the capacity of tumor cells to invade and migrate is not solely dictated by their mesenchymal status but also, to what extent chemotactic signals targeting these cells are present in the tumor microenvironment. With this notion, EMT may be described as a process, which activates tumor cells for invasion and migration, not only by causing loss of cell-cell junctions and reorganization of the cytoskeleton but also by providing tumor cells with the capacity to migrate towards chemotactic signals. This resembles how immune cells become activated and acquire the capacity to sense and migrate towards chemotactic cues during inflammation.

Conditioned medium from lymphatic endothelial cells stimulated chemotactic invasion of mouse mammary tumor cells and human breast cancer cells with mesenchymal properties. The effect was enhanced in the presence of conditioned medium from lymphatic endothelial cells that had been exposed to TGFβ1, suggesting that TGFβ1 induced the expression of genes in lymphatic endothelial cells that encode chemotactic factors for breast cancer cells with mesenchymal properties. RNA sequencing analysis led to the identification of a cluster of inflammatory genes that were induced in lymphatic endothelial cells upon TGFβ1 exposure. Among these were the chemokines *Cx3cl1*, *Cxcl16* and *Ccl17*, which were considered candidate chemotactic factors for breast cancer cells with mesenchymal properties. Overexpression of CX3CL1 has previously been linked to metastasis and poor prognosis in breast cancer [31]. Experimentally, it was shown that CX3CL1 can promote EMT in breast cancer cells, but this was not promoting metastatic spread to lymph nodes when cells were injected into mice [32]. CXCR6, the receptor for CXCL16, is induced in breast cancer cells by hypoxia, and was found to be expressed in lymph metastasis from breast cancer patients [33]. This indicates potential roles for these chemokines in attracting tumor cells towards the lymphatics. However, the expression of their receptors was not associated with mesenchymal features in human breast cancer cells, suggesting that these chemokines do not specifically target breast cancer cells with mesenchymal properties.

In contrast, the receptors for IL7 and IL15 were overexpressed in breast cancer cells with basal-like, mesenchymal properties, compared to luminal cells. IL7 and IL15 belong to the IL-2 family of cytokines that are known for their roles in T cell maturation and homeostasis [34]. IL7 has previously been found to be expressed in lymphatic endothelial cells and vessels and to be important for lymphatic drainage and lymph node remodeling [35, 36]. Most cancer-related studies on IL7 and IL15 have focused on hematological malignancies [37]. During recent years, there has been an increased interest in the IL-2 cytokine family in cancer immunotherapy [38]. The role of IL7 and IL15 in solid tumors is less clear. IL15 was reported to affect breast cancer metastasis in mouse models by regulating the activity of natural killer cells, T cells and macrophages [39]. Overexpression of IL7 and the IL7R correlates with invasion and poor prognosis in breast cancer [40]. Moreover, recent data show that IL7 is part of a cytokine signature linked to metastasis in breast cancer patients [41].

Both IL7 and IL15 stimulated invasion of MDA-MB-231 cells. IL7 and IL15 also stimulated invasion of TGFβ1-treated, mesenchymal NMuMG cells more efficiently than non-treated, epithelial cells. However, the IL7R and the IL15Ra were not induced during TGFβ1-induced EMT in NMuMG cells, suggesting that other signaling pathways drive the expression of these receptors in basal breast cancer cells. The chemotactic effect of IL7 was more potent than that of IL15 and we therefore tested whether a neutralizing antibody against IL7 could inhibit invasion of MDA-MB-231 cells towards conditioned medium from TGFβ1-exposed lymphatic endothelial cells. Indeed, the presence of a neutralizing antibody against IL7 significantly inhibited invasion of MDA-MB-231 cells towards conditioned medium from TGFβ1-exposed lymphatic endothelial cells.

**Fig. 7:**
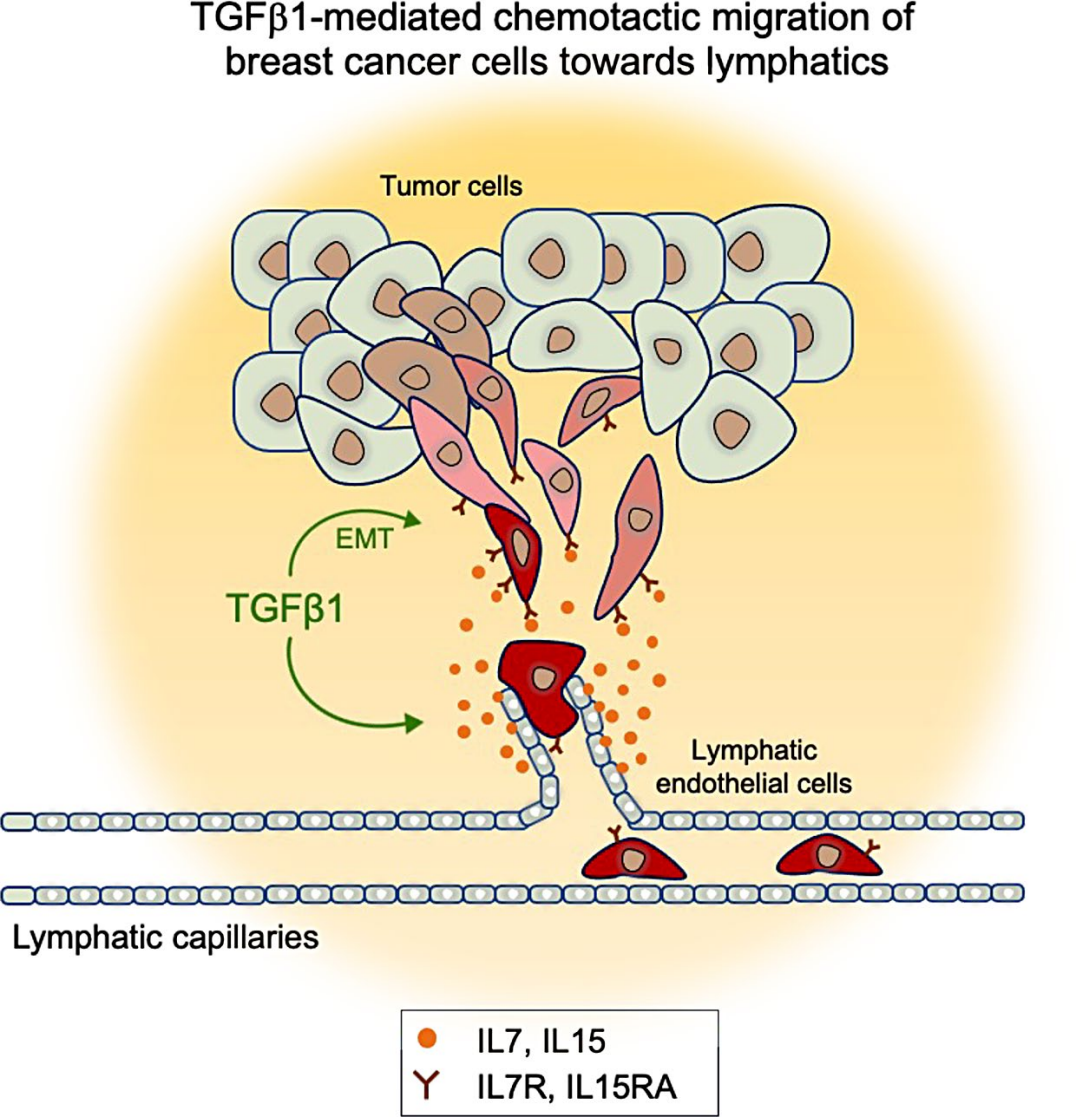
TGFβ1 promotes chemotactic migration of breast cancer cells towards lymphatics. Schematic summary of the results indicating that TGFβ1 plays dual roles in lymphatic dissemination of breast cancer cells by (i) inducing EMT and providing tumor cells with mesenchymal and invasive properties and (ii) stimulating lymphatic endothelial cells to produce chemotactic factors including IL7 and IL15. As a result, breast cancer cells with mesenchymal properties, which overexpress IL7 and IL15 receptors, are activated for chemotactic migration towards the lymphatics

Together, the results indicate that TGFβ1, in addition to its capacity to induce EMT in breast cancer cells, can act on lymphatic endothelial cells to promote the expression of factors, which are chemotactic for breast cancer cells with mesenchymal properties (Fig. 7). Thus, TGFβ1 could play dual roles in lymph metastasis by (i) inducing EMT and thereby providing tumor cells with invasive properties and capabilities to migrate towards chemotactic cues in the tumor microenvironment and (ii) stimulating lymphatic endothelial cells to produce chemotactic factors, which favors chemotactic migration towards the lymphatics. This adds to the previously established pleiotropic role of TGFβ1 as a cytokine, which orchestrates cellular crosstalk and plasticity in the tumor microenvironment. Further analysis are warranted to determine the contribution of IL7 and IL15 in mediating lymphatic dissemination of breast cancer cells in vivo.

### Electronic Supplementary Material

Below is the link to the electronic supplementary material.

Supplementary Material 1
