# Supplementary material for: TGFß1 Stimulates Lymphatic Endothelial Cells to Produce IL7 and IL15, Which Act as Chemotactic Factors for Breast Cancer Cells with Mesenchymal Properties
Source: J Mammary Gland Biol Neoplasia. 2023 Dec 6;28(1):25. doi: 10.1007/s10911-023-09552-y (PMC10700205; doi:10.1007/s10911-023-09552-y)
Supplement: Supplementary file 1 — Supplementary Material 1 [file 10911_2023_9552_MOESM1_ESM.docx]

# TGFβ1 stimulates lymphatic endothelial cells to produce IL7 and IL15, which act as chemotactic factors for breast cancer cells with mesenchymal properties

**Nikolina Giotopoulou^1^, Wenyang Shi^1^, Malgorzata Maria Parniewska^1^, Wenwen Sun^2, 3^, Jonas Fuxe^1, 3, *^**

^1^Karolinska Institutet, Department of Laboratory Medicine, Division of Pathology, SE-14186,

Stockholm, Sweden

^2^Karolinska Institutet, Department of Oncology-Pathology, SE-17164, Stockholm, Sweden

^3^Division of Clinical Pathology and Cancer Diagnostics, Karolinska University Laboratory, Karolinska University Hospital, SE-14186, Stockholm, Sweden

***Correspondence:** Jonas Fuxe, [Jonas.fuxe@ki.se](mailto:Jonas.fuxe@ki.se)


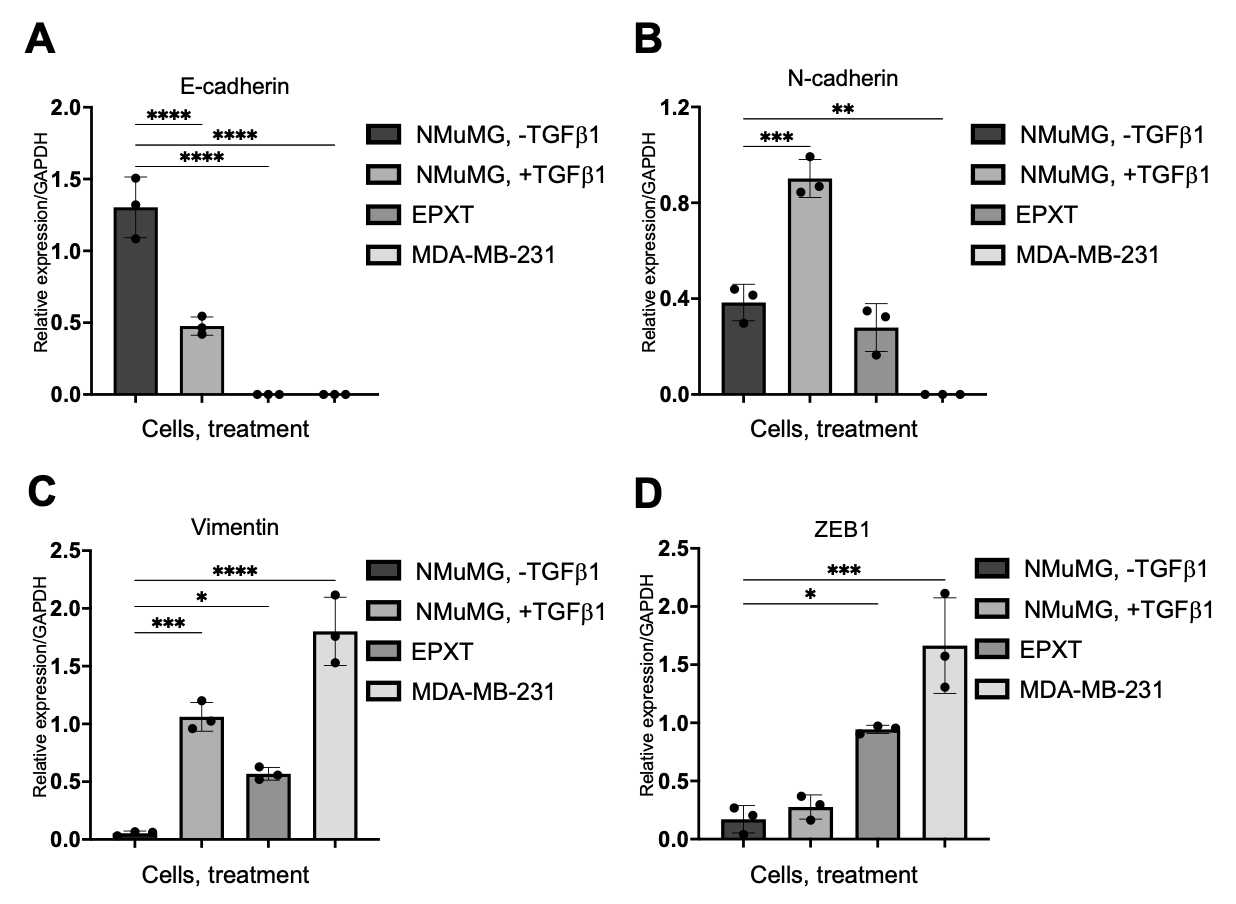


**Figure S1. Quantification of Western blot data in Fig. 1A. (A-F)** Graphs showing protein expression of EMT markers in untreated (control) and TGFβ1 treated NMuMG cells, EpXT cells and MDA-MB-231 (MDA231) cells. Data represent results from three independent experiments with three technical replicates per condition and experiment. **** = P < 0.0001, *** = P < 0.001, ** = P < 0.01, * = P < 0.05.


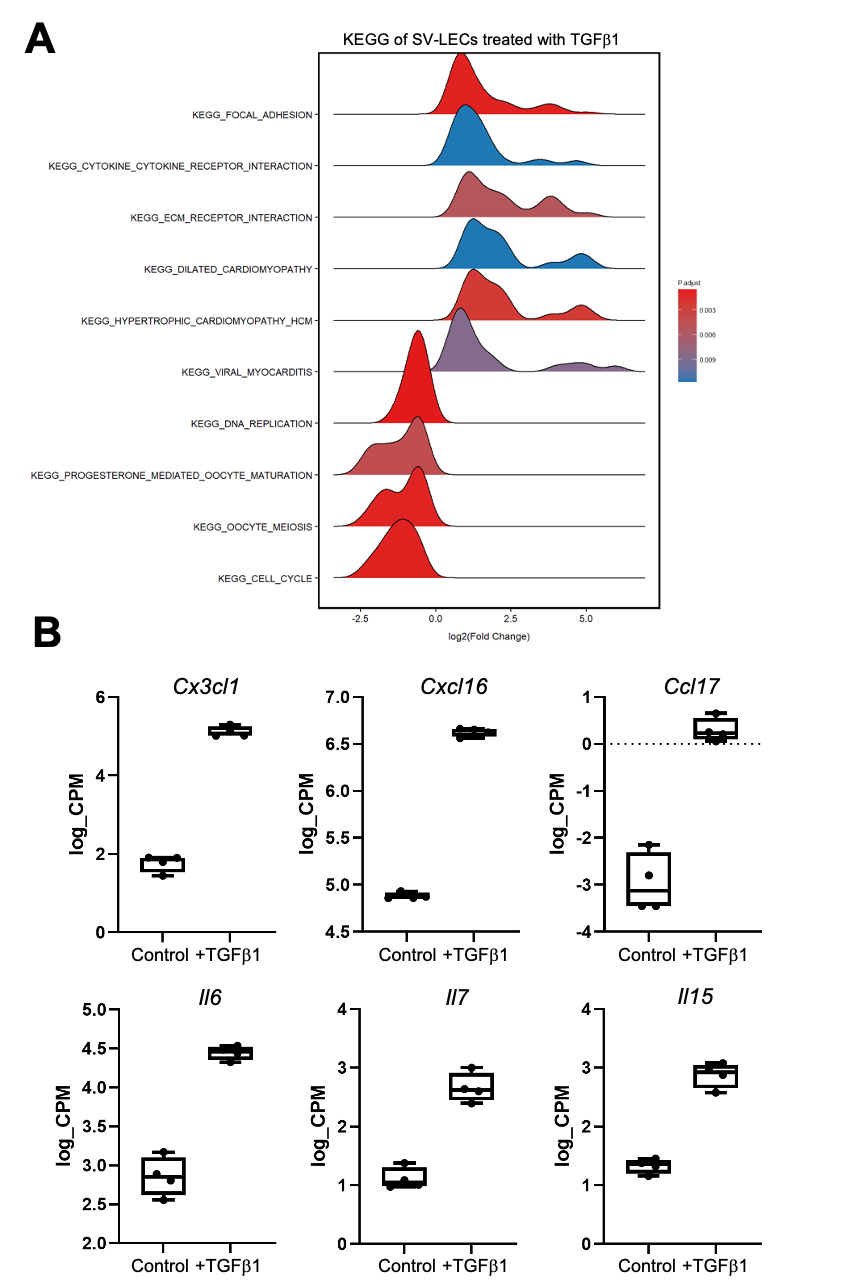


**Figure S2. Analysis of the effect of TGFβ1 on gene expression in SV-LECs. (A)** Gene set Enrichment analysis of molecular signature database and KEGG pathways. (**B**) Data from RNA seq analysis showing differences in expression values (log_CPM) of the chemokines *Cx3cl1*, *Cxcl16* and *Ccl17*, and the interleukins *Il6*, *Il7* and *Il15* in TGFβ1 treated compared to non-treated (control) SV-LEC lymphatic endothelial cells.


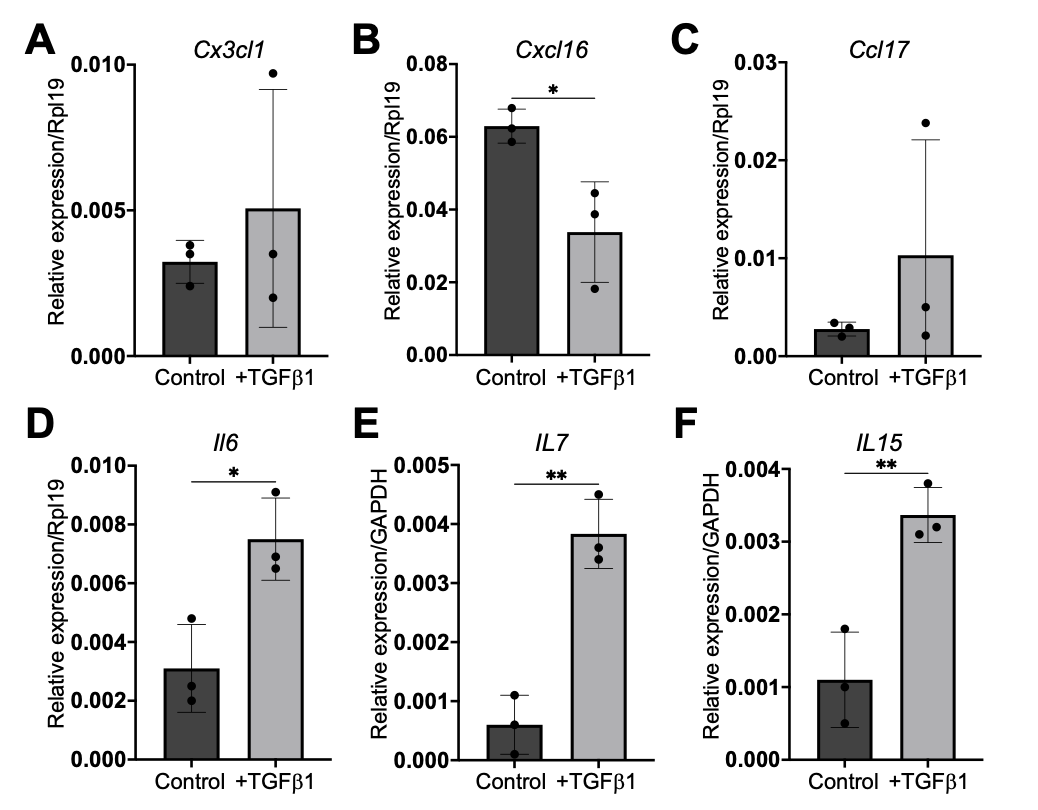


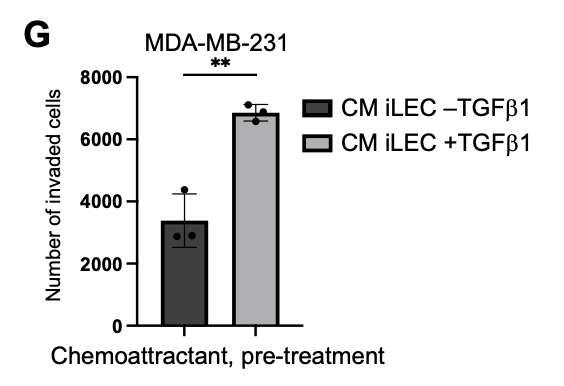


**Figure S3. Changes in chemokine expression in TGFβ1 treated versus non-treated lymphatic endothelial cells.** (**A-D**) QPCR results showing the expression of chemokines in untreated (control) and TGFβ1 treated SV-LEC cells. (**E, F**) QPCR results showing the expression of *IL7* and *IL15* in untreated (control) and TGFβ1 treated human iLEC cells. Data points represent technical replicates from three independent qPCR experiments. (**G**) Results from invasion assays showing that conditioned medium from iLEC cells exposed to TGFβ1 stimulates migration of MDA-MB-231 cells more than medium from non-treated cells. Data points represent averages from three independent qPCR experiments. **** = P < 0.0001; ** = P < 0.01; * = P < 0.05.


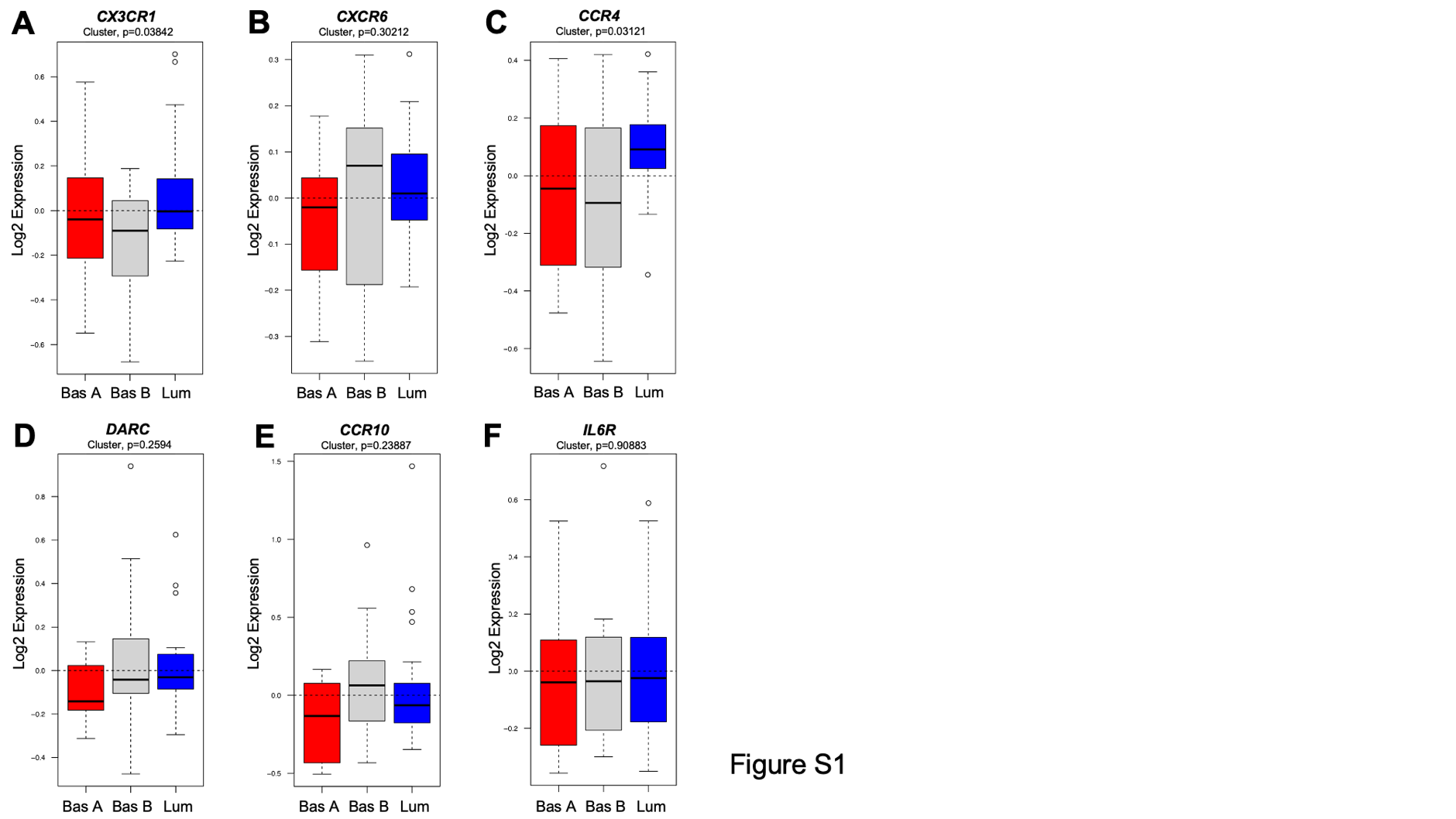


**Figure S4. Log2 expression data from the GOBO database. (A-F)** Graphs showing the expression of *CX3CR1* (**A**), *CXCR6* (**B**), *CCR4* (**C**), *DARC* (**D**), *CCR10* (**E**) and *IL6R* (**F**), in human breast cancer cells classified as basal A (Bas A, red bars), basal B (Bas B, grey bars) or luminal cells (Lum, blue bars).


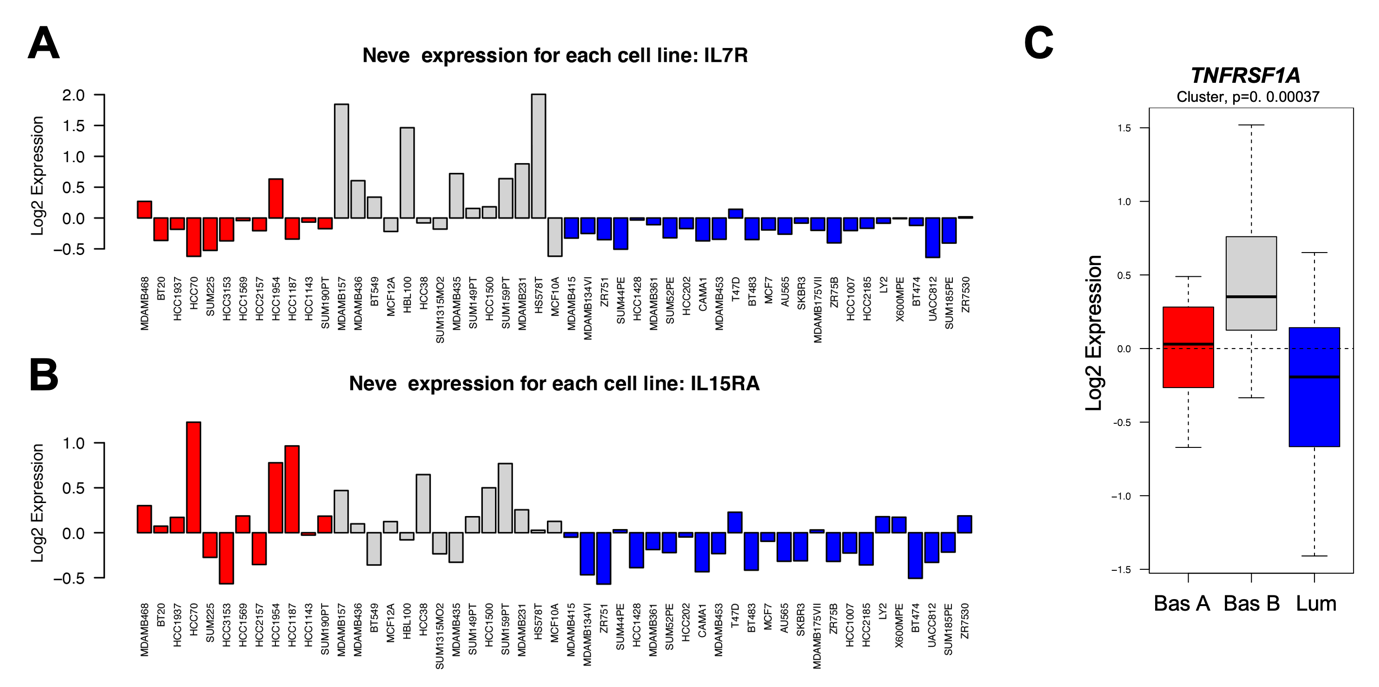


***IL7R* expression in human breast cancer cells**

***IL15RA* expression in human breast cancer cells**


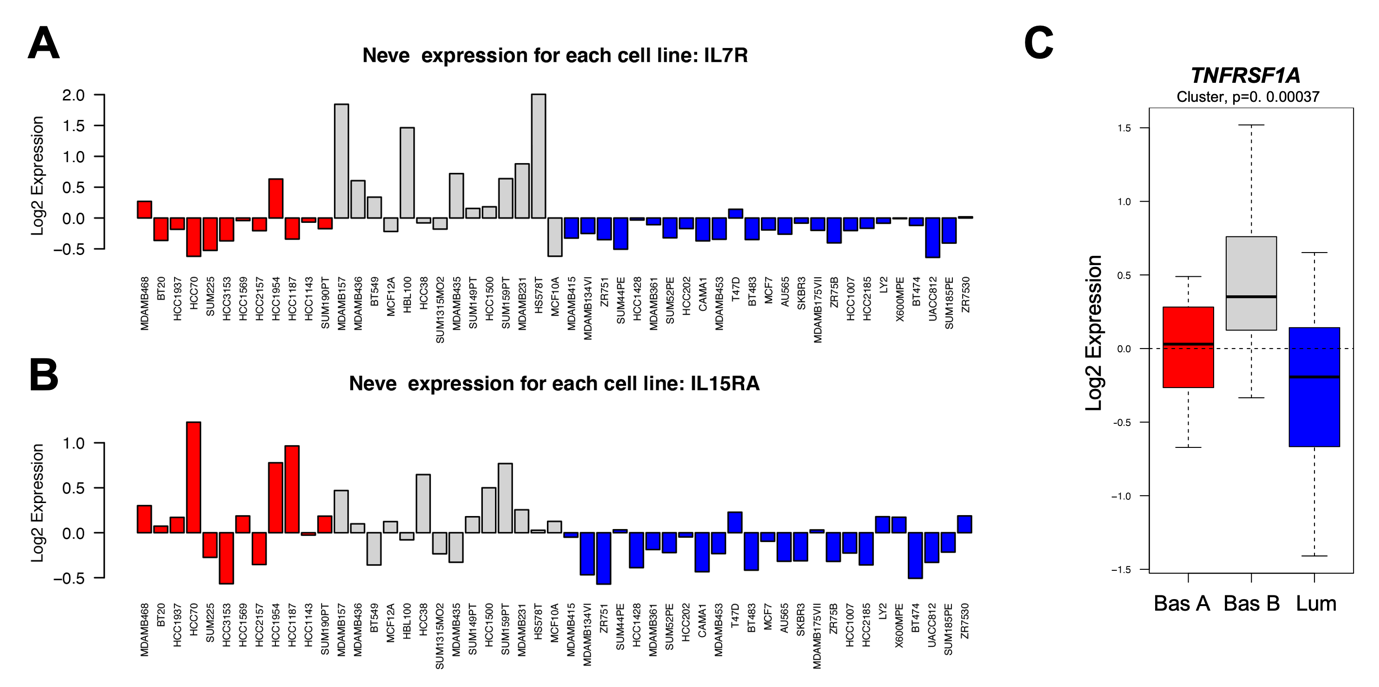


**Figure S5. Log2 expression data from the**

**GOBO database. (A, B)** Log2 expression of

the *IL7R* (**A**) and *IL15RA* (**B**) in individual

human breast cancer cells classified as basal A

(red), basal B (grey) or luminal cells (blue).

(**C**) Log2 expression of *TNFRSF1A* in human

breast cancer cells classified as basal A (red),

basal B (grey) or luminal cells (blue).

**Supplementary Table 1:** **List of primers used for qPCR.**

**
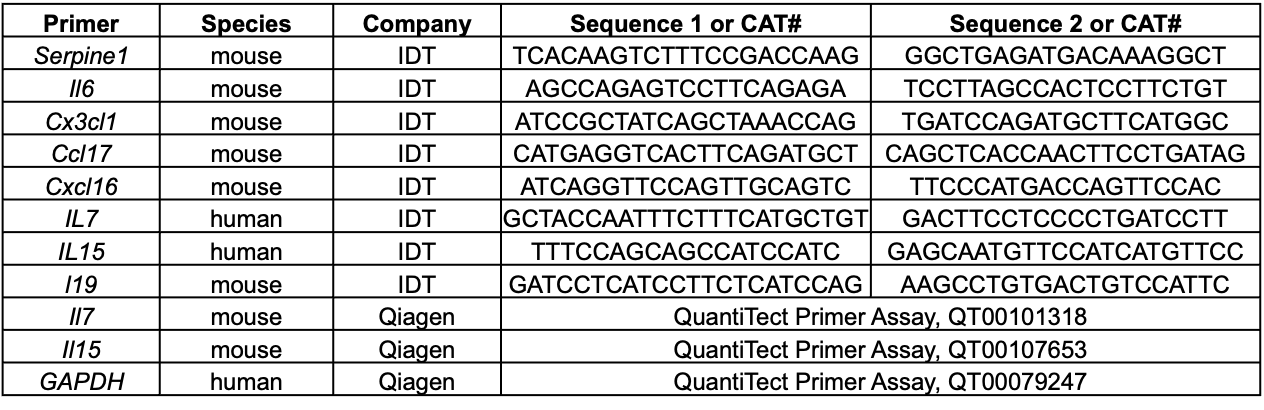
**

**Supplementary Table 2:** List of 120 genes induced >1.5logFC in SV-LEC cells by TGFβ1 and belonging to GO term: extracellular space.

| **ID** | **Gene** | **Protein** | **logFC** | **FDR** |
| --- | --- | --- | --- | --- |
| ENSMUSG00000033453 | *Adamts15* | A disintegrin-like and metallopeptidase (reprolysin type) with thrombospondin type 1 motif, 15 | 4.308429769 | 1.6863E-57 |
| ENSMUSG00000034730 | *Adgrb1* | Adhesion G protein-coupled receptor B1 | 6.266719024 | 1.10807E-31 |
| ENSMUSG00000015452 | *Ager* | Advanced glycosylation end product-specific receptor | 1.780792673 | 1.08999E-22 |
| ENSMUSG00000030607 | *Acan* | Aggrecan | 7.161630502 | 6.6652E-132 |
| ENSMUSG00000029378 | *Areg* | Amphiregulin | 2.698196826 | 2.7898E-24 |
| ENSMUSG00000027460 | *Angpt4* | Angiopoietin 4 | 1.801156593 | 3.74168E-43 |
| ENSMUSG00000004105 | *Angptl2* | Angiopoietin-like 2 | 2.172098679 | 1.9442E-192 |
| ENSMUSG00000002289 | *Angptl4* | Angiopoietin-like 4 | 3.177131188 | 0 |
| ENSMUSG00000056656 | *Apol8* | Apolipoprotein L 8 | 3.505050036 | 7.56776E-60 |
| ENSMUSG00000074483 | *Bglap* | Bone gamma carboxyglutamate protein | 3.319013215 | 9.60033E-21 |
| ENSMUSG00000022098 | *Bmp1* | Bone morphogenetic protein 1 | 1.965381611 | 0 |
| ENSMUSG00000032726 | *Bmp8a* | Bone morphogenetic protein 8a | 3.414556302 | 2.1725E-106 |
| ENSMUSG00000058914 | *C1qtnf3* | C1q and tumor necrosis factor related protein 3 | 3.620867807 | 1.9188E-242 |
| ENSMUSG00000003814 | *Calr* | Calreticulin | 1.506542061 | 1.0352E-237 |
| ENSMUSG00000056737 | *Capg* | Capping protein (actin filament), gelsolin-like | 1.782436391 | 5.2396E-138 |
| ENSMUSG00000036596 | *Cpz* | Carboxypeptidase Z | 1.989129974 | 0 |
| ENSMUSG00000044006 | *Cilp2* | Cartilage intermediate layer protein 2 | 2.051633559 | 5.39404E-21 |
| ENSMUSG00000083282 | *Ctsf* | Cathepsin F | 1.684838191 | 3.1529E-256 |
| ENSMUSG00000024910 | *Ctsw* | Cathepsin W | 2.186545267 | 1.89611E-79 |
| ENSMUSG00000019997 | *Ccn2* | Cellular communication network factor 2 | 2.072427598 | 4.2384E-286 |
| ENSMUSG00000027656 | *Ccn5* | Cellular communication network factor 5 | 5.320465903 | 0 |
| ENSMUSG00000031780 | *Ccl17* | Chemokine (C-C motif) ligand 17 | 4.554454169 | 1.85908E-24 |
| ENSMUSG00000031778 | *Cx3cl1* | Chemokine (C-X3-C motif) ligand 1 | 3.461111029 | 5.069E-228 |
| ENSMUSG00000018920 | *Cxcl16* | Chemokine (C-X-C motif) ligand 16 | 1.825047258 | 5.326E-287 |
| ENSMUSG00000020953 | *Coch* | Cochlin | 1.675790478 | 9.27882E-10 |
| ENSMUSG00000046318 | *Ccbe1* | Collagen and calcium binding EGF domains 1 | 1.616367999 | 6.19136E-12 |
| ENSMUSG00000054196 | *Cthrc1* | Collagen triple helix repeat containing 1 | 2.464313525 | 4.5342E-101 |
| ENSMUSG00000022483 | *Col2a1* | Collagen, type II, alpha 1 | 4.273663981 | 7.94564E-63 |
| ENSMUSG00000079465 | *Col4a3* | Collagen, type IV, alpha 3 | 2.311852612 | 2.25167E-17 |
| ENSMUSG00000067158 | *Col4a4* | Collagen, type IV, alpha 4 | 1.636875816 | 4.02989E-32 |
| ENSMUSG00000027570 | *Col9a3* | Collagen, type IX, alpha 3 | 2.362255622 | 2.2568E-63 |
| ENSMUSG00000004098 | *Col5a3* | Collagen, type V, alpha 3 | 1.805005116 | 1.14138E-93 |
| ENSMUSG00000025650 | *Col7a1* | Collagen, type VII, alpha 1 | 2.220355396 | 7.47041E-22 |
| ENSMUSG00000039462 | *Col10a1* | Collagen, type X, alpha 1 | 3.721651208 | 8.20697E-24 |
| ENSMUSG00000027966 | *Col11a1* | Collagen, type XI, alpha 1 | 3.960515481 | 0 |
| ENSMUSG00000025064 | *Col17a1* | Collagen, type XVII, alpha 1 | 5.407061262 | 3.22497E-36 |
| ENSMUSG00000001435 | *Col18a1* | Collagen, type XVIII, alpha 1 | 2.014654519 | 5.2389E-285 |
| ENSMUSG00000016356 | *Col20a1* | Collagen, type XX, alpha 1 | 1.858516905 | 9.26384E-38 |
| ENSMUSG00000045672 | *Col27a1* | Collagen, type XXVII, alpha 1 | 1.905116922 | 2.78018E-60 |
| ENSMUSG00000057606 | *Colq* | Collagen-like tail subunit (single strand of homotrimer) of asymmetric acetylcholinesterase | 3.141889768 | 1.0424E-32 |
| ENSMUSG00000015451 | *C4a* | Complement component 4A (Rodgers blood group) | 2.255154576 | 4.88214E-18 |
| ENSMUSG00000001128 | *Cfp* | Complement factor properdin | 1.760438583 | 1.38948E-26 |
| ENSMUSG00000001270 | *Ckb* | Creatine kinase, brain | 1.648571608 | 2.09671E-28 |
| ENSMUSG00000031825 | *Crispld2* | Cysteine-rich secretory protein LCCL domain containing 2 | 2.226715645 | 6.2702E-171 |
| ENSMUSG00000007888 | *Crlf1* | Cytokine receptor-like factor 1 | 4.945004661 | 6.3924E-65 |
| ENSMUSG00000044258 | *Ctla2a* | Cytotoxic T lymphocyte-associated protein 2 alpha | 2.792336804 | 0 |
| ENSMUSG00000074874 | *Ctla2b* | Cytotoxic T lymphocyte-associated protein 2 beta | 1.586618141 | 3.18653E-08 |
| ENSMUSG00000037370 | *Enpp1* | Ectonucleotide pyrophosphatase/phosphodiesterase 1 | 2.4603145 | 1.6329E-223 |
| ENSMUSG00000029377 | *Ereg* | Epiregulin | 2.53179094 | 1.498E-249 |
| ENSMUSG00000028108 | *Ecm1* | Extracellular matrix protein 1 | 2.015074218 | 0 |
| ENSMUSG00000020614 | *Fam20a* | FAM20A, golgi associated secretory pathway pseudokinase | 2.198276316 | 6.1914E-153 |
| ENSMUSG00000028773 | *Fabp3* | Fatty acid binding protein 3, muscle and heart | 1.615676541 | 4.03095E-09 |
| ENSMUSG00000039899 | *Fgl2* | Fibrinogen-like protein 2 | 1.539117692 | 1.19474E-07 |
| ENSMUSG00000057967 | *Fgf18* | Fibroblast growth factor 18 | 1.543150738 | 7.35626E-37 |
| ENSMUSG00000021765 | *Fst* | Follistatin | 1.949314844 | 0 |
| ENSMUSG00000020325 | *Fstl3* | Follistatin-like 3 | 1.585933349 | 2.8E-70 |
| ENSMUSG00000024907 | *Gal* | Galanin and GMAP prepropeptide | 3.437446129 | 7.24902E-26 |
| ENSMUSG00000056888 | *Glipr1* | GLI pathogenesis-related 1 (glioma) | 1.951394646 | 2.83229E-30 |
| ENSMUSG00000024784 | *Gpha2* | Glycoprotein hormone alpha 2 | 1.821254809 | 1.54642E-10 |
| ENSMUSG00000021943 | *Gdf10* | Growth differentiation factor 10 | 1.780303619 | 8.46286E-28 |
| ENSMUSG00000072625 | *Gdf2* | Growth differentiation factor 2 | 1.540192078 | 4.95088E-09 |
| ENSMUSG00000018102 | *H2bc4* | H2B clustered histone 4 | 2.916105528 | 4.12591E-60 |
| ENSMUSG00000047246 | *H2bc6* | H2B clustered histone 6 | 5.8017635 | 5.8417E-128 |
| ENSMUSG00000055632 | *Hmcn2* | Hemicentin 2 | 2.156771133 | 1.4018E-92 |
| ENSMUSG00000029102 | *Hgfac* | Hepatocyte growth factor activator | 4.064152302 | 1.04997E-51 |
| ENSMUSG00000073409 | *H2-Q6* | Histocompatibility 2, Q region locus 6 | 1.952707523 | 1.85229E-61 |
| ENSMUSG00000010051 | *Hyal1* | Hyaluronoglucosaminidase 1 | 2.896057267 | 8.369E-238 |
| ENSMUSG00000037035 | *Inhbb* | Inhibin beta-B | 2.047646344 | 1.2298E-152 |
| ENSMUSG00000036256 | *Igfbp7* | Insulin-like growth factor binding protein 7 | 2.115965077 | 1.6278E-270 |
| ENSMUSG00000031712 | *Il15* | Interleukin 15 | 1.653192778 | 7.08508E-29 |
| ENSMUSG00000025746 | *Il6* | Interleukin 6 | 1.652534454 | 6.20867E-51 |
| ENSMUSG00000040329 | *Il7* | Interleukin 7 | 1.672925582 | 2.65318E-23 |
| ENSMUSG00000023043 | *Krt18* | Keratin 18 | 2.483785587 | 2.78508E-12 |
| ENSMUSG00000035237 | *Lcat* | Lecithin cholesterol acyltransferase | 2.500179709 | 8.93603E-67 |
| ENSMUSG00000053964 | *Lgals4* | Lectin, galactose binding, soluble 4 | 1.922787802 | 5.95114E-22 |
| ENSMUSG00000052316 | *Lrrc15* | Leucine rich repeat containing 15 | 5.518860369 | 6.72851E-37 |
| ENSMUSG00000090958 | *Lrrc32* | Leucine rich repeat containing 32 | 2.574233303 | 1.6777E-272 |
| ENSMUSG00000042793 | *Lgr6* | Leucine-rich repeat-containing G protein-coupled receptor 6 | 3.01996056 | 1.73924E-28 |
| ENSMUSG00000044626 | *Liph* | Lipase, member H | 2.91015831 | 6.74535E-18 |
| ENSMUSG00000000693 | *Loxl3* | Lysyl oxidase-like 3 | 1.717846304 | 1.2351E-291 |
| ENSMUSG00000032591 | *Mst1* | Macrophage stimulating 1 (hepatocyte growth factor-like) | 4.126839347 | 1.45485E-21 |
| ENSMUSG00000022887 | *Masp1* | Mannan-binding lectin serine peptidase 1 | 2.576705082 | 8.53986E-36 |
| ENSMUSG00000005142 | *Man2b1* | Mannosidase 2, alpha B1 | 2.783330471 | 0 |
| ENSMUSG00000017737 | *Mmp9* | Matrix metallopeptidase 9 | 5.286470604 | 1.3922E-158 |
| ENSMUSG00000032135 | *Mcam* | Melanoma cell adhesion molecule | 2.608343285 | 0 |
| ENSMUSG00000058183 | *Mmel1* | Membrane metallo-endopeptidase-like 1 | 2.867935613 | 7.59289E-33 |
| ENSMUSG00000042436 | *Mfap4* | Microfibrillar-associated protein 4 | 1.59438793 | 1.15295E-07 |
| ENSMUSG00000041445 | *Mmrn2* | Multimerin 2 | 2.862080374 | 1.82874E-65 |
| ENSMUSG00000020826 | *Nos2* | Nitric oxide synthase 2, inducible | 2.102022512 | 4.93615E-25 |
| ENSMUSG00000026833 | *Olfm1* | Olfactomedin 1 | 2.580845204 | 7.20805E-19 |
| ENSMUSG00000032172 | *Olfm2* | Olfactomedin 2 | 1.873161667 | 1.94987E-44 |
| ENSMUSG00000017754 | *Pltp* | Phospholipid transfer protein | 2.907493446 | 0 |
| ENSMUSG00000004791 | *Pgf* | Placental growth factor | 3.299541877 | 1.10475E-38 |
| ENSMUSG00000028019 | *Pdgfc* | Platelet-derived growth factor, C polypeptide | 1.614860932 | 2.40301E-64 |
| ENSMUSG00000012889 | *Podnl1* | Podocan-like 1 | 2.051548505 | 6.21278E-38 |
| ENSMUSG00000028370 | *Pappa* | Pregnancy-associated plasma protein A | 1.993684595 | 4.74023E-30 |
| ENSMUSG00000078949 | *R3hdml* | R3H domain containing-like | 3.727979205 | 2.99014E-20 |
| ENSMUSG00000078776 | *9530053A07Rik* | RIKEN cdna 9530053A07 gene | 2.360970435 | 2.80069E-88 |
| ENSMUSG00000035279 | *Ssc5d* | Scavenger receptor cysteine rich family, 5 domains | 2.996772232 | 2.1034E-180 |
| ENSMUSG00000021904 | *Sema3g* | Sema domain, immunoglobulin domain (Ig), short basic domain, secreted, (semaphorin) 3G | 1.903641265 | 4.91113E-11 |
| ENSMUSG00000019647 | *Sema6a* | Sema domain, transmembrane domain (TM), and cytoplasmic domain, (semaphorin) 6A | 2.081882901 | 1.45618E-10 |
| ENSMUSG00000044734 | *Serpinb1a)* | Serine (or cysteine) peptidase inhibitor, clade B, member 1a | 2.784952946 | 9.3758E-126 |
| ENSMUSG00000037411 | *Serpine1* | Serine (or cysteine) peptidase inhibitor, clade E, member 1 | 1.669733798 | 0 |
| ENSMUSG00000026249 | *Serpine2* | Serine (or cysteine) peptidase inhibitor, clade E, member 2 | 3.206965723 | 0 |
| ENSMUSG00000027834 | *Serpini1* | Serine (or cysteine) peptidase inhibitor, clade I, member 1 | 2.198815829 | 6.9733E-291 |
| ENSMUSG00000025020 | *Slit1* | Slit guidance ligand 1 | 3.302955945 | 1.97573E-88 |
| ENSMUSG00000021136 | *Smoc1* | SPARC related modular calcium binding 1 | 2.270490954 | 6.80323E-36 |
| ENSMUSG00000023886 | *Smoc2* | SPARC related modular calcium binding 2 | 2.069089072 | 1.44955E-20 |
| ENSMUSG00000072941 | *Sod3* | Superoxide dismutase 3, extracellular | 1.651859062 | 1.97601E-60 |
| ENSMUSG00000031995 | *St14* | Suppression of tumorigenicity 14 (colon carcinoma) | 3.891934548 | 0 |
| ENSMUSG00000040152 | *Thbs1* | Thrombospondin 1 | 2.402856812 | 0 |
| ENSMUSG00000053626 | *Tll1* | Tolloid-like | 1.933486469 | 8.40983E-87 |
| ENSMUSG00000029999 | *Tgfa* | Transforming growth factor alpha | 1.64928679 | 6.20457E-24 |
| ENSMUSG00000063727 | *Tnfrsf11b* | Tumor necrosis factor receptor superfamily, member 11b (osteoprotegerin) | 3.149444599 | 4.2499E-132 |
| ENSMUSG00000031520 | *Vegfc* | Vascular endothelial growth factor C | 3.699330465 | 2.5949E-102 |
| ENSMUSG00000037428 | *Vgf* | VGF nerve growth factor inducible | 2.824569037 | 5.03082E-18 |
| ENSMUSG00000071192 | *Wfikkn1* | WAP, FS, Ig, KU, and NTR-containing protein 1 | 2.369690286 | 3.69263E-30 |
| ENSMUSG00000036856 | *Wnt4* | Wingless-type MMTV integration site family, member 4 | 3.706057359 | 1.1777E-207 |
| ENSMUSG00000022382 | *Wnt7b* | Wingless-type MMTV integration site family, member 7B | 1.636891681 | 5.08632E-12 |
| ENSMUSG00000000126 | *Wnt9a* | Wingless-type MMTV integration site family, member 9A | 2.009512207 | 4.3896E-188 |
